# Electric vehicle braking energy recovery control method integrating fuzzy control and improved firefly algorithm

**DOI:** 10.1371/journal.pone.0320537

**Published:** 2025-03-28

**Authors:** Jinfeng Xiong, Jingbin Song, Zhiqiang Zhang

**Affiliations:** College of Transportation Engineering, Changzhou Vocational Institute of Mechatronic Technology, Changzhou, China; Beijing University of Technology, CHINA

## Abstract

Braking energy recovery is crucial for improving the energy efficiency and extending the range of electric vehicles. If a large amount of braking energy is wasted, it will lead to problems such as reduced range and increased battery burden for electric vehicles. Therefore, an electric vehicle braking energy recovery control model that integrates fuzzy control algorithm with genetic firefly algorithm is proposed. Experimental analysis showed that the decrease in the state of charge of the model was 12.44%, and the braking energy recovery rate reached 52.1% in practical applications. Based on the above data, the proposed method can effectively control the amount of energy recovery. In addition, when the system chip value was 10%, the total amount of recovered energy at the battery end was the highest. Conversely, the total amount of recovered energy at the battery end was relatively small. In summary, the designed electric vehicle braking energy recovery control model can effectively control the amount of braking energy recovery of electric vehicles, ensuring the maximum recovery while also considering the durability and driving stability of the vehicle battery. The method can effectively extend mileage range in the electric vehicle industry, promoting the development and technological innovation of the new energy industry.

## 1. Introduction

Affected by the new energy vehicle market, the construction and layout of electric vehicle charging facilities have also been continuously promoted. New energy vehicles benefit from the development of battery technology. The Institute of Physics of the Chinese Academy of Sciences has developed a solid state lithium battery with an energy density of 360Wh/kg, which marks an important step in the field of electric vehicles in China [1]. As of the end of May 2023, the charging infrastructure has reached 6.356 million units, of which public charging stations account for 33% and private charging stations account for 67%. More people are choosing to purchase electric vehicles [2]. China’s electric vehicles have firmly established a world leading position in the “three electric” system of batteries, motors, and electronic control, which is also a symbol of key technological breakthroughs [3]. However, electric vehicles also have some drawbacks, such as mileage anxiety, slow charging, and pollution from used batteries. The battery life is a serious problem faced by the electric vehicle industry, which directly determines consumers’ choice. At present, the common mileage of electric vehicles on the market is 600km, which is significantly different from that of gasoline vehicles. Moreover, 600km is the optimal mileage under normal driving conditions, and the mileage is greatly weakened in harsh environments [4,5]. Therefore, the electric vehicle industry urgently needs to optimize the range issue. Related research has focused on the amount of Braking Energy Recovery (BER), while electric vehicle BER requires effective control of energy flow.

Electric vehicle is an effective way to respond to energy and environmental problems, but its limited range severely restricts the promotion and application of electric vehicles. Recovery braking technology is a crucial means to improve the driving mileage of electric vehicles. Biao J et al. designed a BER strategy based on optimization allocation algorithm for energy recovery. It optimized the braking force allocation between the front and rear axles in a variable proportion according to the braking intensity. This strategy could improve the BER efficiency to over 51.9% while maintaining braking stability [6]. Armenta-DéuC et al. analyzed the kinetic energy recovery of electric vehicles. Two mainstream braking energy recovery systems were analyzed: regenerative braking and potential energy conversion. The results indicated that potential energy was a potential source of kinetic energy recovery, which was more efficient than traditional regenerative braking systems. The maximum efficiency achieved by the regenerative braking system was 60.1% [7]. Jamadar N M et al. proposed a BER system to address the friction caused by mechanical braking mechanisms, which was converted into heat loss and reduced the efficiency of electric vehicles. The combined control system could adapt to the BER efficiency of different electric vehicles [8]. Guo J et al. found that intelligent electric vehicles were receiving increasing attention due to their higher safety and energy efficiency. Therefore, an adaptive cruise control strategy that considered recovery braking was proposed to improve the safety and energy efficiency of electric vehicles. The control method had good longitudinal tracking and BER performance without sacrificing safety [9]. Guo B et al. proposed a friction heat recovery system based on a heavy-duty vehicle hydraulic line control dynamic system to reduce environmental pollution caused by excessive waste of braking energy. The system used the thermal energy generated by friction to generate electricity and stored the electrical energy in the vehicle battery. The results showed that the braking energy consumption of the system was reduced, and the fuel consumption was decreased by 10% compared to before [10].

The braking energy control algorithm has been widely discussed both domestically and internationally regarding the method to energy recovery control in electric vehicles. Pan Y et al. found that the existing results based on mixed triggering mechanisms had complex control structures. Therefore, a non-linear network control strategy based on uncertain event triggered variables was designed for the first time. This method could stably transmit key information to the controller under non-periodic network attacks [11]. Mei P et al. proposed a novel fuzzy sliding mode control scheme under adaptive control strategy for energy management mechanism of electric vehicles constrained by regenerative braking system. The results showed that this scheme could integrate the optimal battery state and energy recovery efficiency [12]. Wang Y et al. built a fuzzy static output feedback controller that relied on patterns and changes for the control and filtering problems of Markov jump singular perturbation systems. The results showed that the fuzzy control was feasible and effective [13]. Zare M et al. found that the motion process of the Firefly Algorithm (FA) was not obvious. Therefore, a differential evolution and current optimization was proposed to optimize the global search ability and convergence speed. This method was effective and robust in enhancing the FA [14]. Li X et al. found that road slope angle had a significant impact on braking stability and braking energy recovery efficiency. Therefore, a method based on vehicle fixed ratio and variable ratio braking force allocation was proposed. The results showed that these two strategies could ensure the braking stability of the vehicle. As the slope angle increased, the recovered energy significantly increased [15]. The advantages and disadvantages of the research method for electric vehicle braking in the literature review are compared in Table 1.

**Table 1:** Comparison of advantages and disadvantages of literature review.

Method	Research contents	Advantage	Disadvantage	Referen-ces
Regenerative braking control strategy for electric vehicles based on braking stability requirements	Optimize the distribution of braking force between the front and rear axles through variable proportions. Compared with the FC-FA method, both methods focus on optimizing the allocation in braking to improve the efficiency of braking energy recovery.	Being able to optimize the brake force distribution between the front and rear axles in a variable proportion based on the braking intensity, and improving the braking energy recovery efficiency to over 51.9%, which helps to increase the range of electric vehicles.	Complex sensors and control systems may be required to accurately identify braking intensity and optimize real-time braking force distribution.	Biao J et al [6]
Two mainstream braking energy recovery systems: regenerative braking and potential energy conversion.	By comparing the use of potential energy conversion and regenerative braking, it was found that the efficiency of potential energy conversion is higher. Compared with the FC-FA method, both emphasize how to maintain braking stability and driving safety.	The research suggests that potential energy conversion, as a potential source of energy, may have higher efficiency than traditional regenerative braking systems.	Under certain driving conditions or terrains, the potential energy conversion system may not be as effective as the regenerative brake system.	Armenta-Déu C et al [7]
A review of current research on brake control and optimization technology for electric vehicles	Improve efficiency by reducing the heat loss of mechanical braking. Improve efficiency by reducing the heat loss of mechanical braking. Similar to the goal of FC-FA, both aim to adapt to energy recovery requirements under different operating conditions through algorithm optimization.	By reducing the frictional heat loss caused by mechanical braking mechanisms, the efficiency of electric vehicles is improved.	Significant modifications are required to the existing braking system, which increases costs and implementation difficulties.	Jamadar N M et al [8]
A safety and energy efficiency following control strategy for intelligent electric vehicles considering regenerative braking	The study aims to achieve brake energy recovery through an adaptive cruise control framework, while the FC-FA method improves energy recovery efficiency by optimizing brake force distribution.	Improved the safety and energy efficiency of electric vehicles, with good longitudinal tracking and braking energy recovery performance.	The implementation of adaptive cruise control system may require advanced sensors and complex control algorithms, which require high reliability and robustness of the system.	Guo J et al [9]
Friction heat recovery system based on heavy-duty vehicle hydraulic line control dynamic system	The study converts frictional heat energy into electrical energy and stores it in batteries, while the FC-FA method improves braking energy recovery efficiency by optimizing braking force distribution	Utilizing frictional heat energy to generate electricity is an innovative method that provides new possibilities for energy recovery in heavy-duty vehicles.	This system involves complex technological implementation, including thermal energy conversion and storage technology.	Guo B et al [10]
FC-FA	Electric Vehicle Braking Energy Recovery Control Model Integrating Fuzzy Control Algorithm and Genetic Firefly Algorithm	Simultaneously considering multiple objectives such as energy recovery efficiency, braking stability, and economy, and improving overall performance through multi-objective optimization.	Genetic algorithms and fuzzy control may require more computing resources, especially in real-time systems, which may have higher performance requirements for processors.	This study

In summary, although existing literature can help further understand the relevant theories of electric vehicle braking energy recovery, there are also complexities that have not been taken into account in practical applications, such as irregular braking behavior, different driving styles, and changes in road types. This may result in existing control strategies not achieving the expected performance in practical applications and failing to meet the requirements of precise control. Meanwhile, a single algorithm has problems such as long computation time and low decoding efficiency when calculating the recovered energy. Based on this, the study proposes to improve the FA by combining it with genetic algorithm. Then, the optimized FA is integrated with Fuzzy Logic Control (FC) algorithm to construct an FC-FA electric vehicle braking energy recovery model to ensure driving safety while achieving maximum braking energy recovery. Compared with existing related methods, fuzzy control is used to handle system uncertainty and external disturbances, while genetic algorithm is used to optimize system parameters. Therefore, the novelty of the research lies in the combination of these two methods, which not only improves the system’s adaptability and robustness to different operating conditions, but also optimizes the system parameters to adapt to these changes. In addition, in existing research, energy recovery systems usually adopt fixed recovery strategies and cannot be dynamically adjusted according to different driving conditions and environmental changes. The research innovatively combines the global search capability of genetic algorithms with the local adjustment capability of fuzzy control, achieving more accurate energy recovery management.

Compared with existing related methods, the research has made significant contributions: (1) By reasonably controlling the charging and discharging process of the battery, avoiding overcharging and deep discharge, the service life of the battery can be effectively extended. (2) The research and application of FC-FA model have promoted the development of energy recovery technology for electric vehicle braking, providing technical support for the commercialization and popularization of electric vehicles. This indicates that research not only stays at the theoretical level, but also has a direct driving effect on practical applications and industrial progress. (3) The effective recovery of braking energy can reduce the energy consumption of electric vehicles, lower operating costs, and improve economic efficiency. The effective recovery of braking energy can reduce the energy consumption of electric vehicles, lower operating costs, and improve economic efficiency. (4) The constructed control model involves multiple disciplines such as control theory and energy management, promoting the in-depth development of interdisciplinary research.

## 2. Methods and materials

The study first elaborates on the power distribution method for BER of electric vehicles and proposes the FC algorithm. Subsequently, the calculation equation of FA is proposed and combined with genetic algorithm to improve the original FA. Finally, a new FC-FA control model is constructed by combining the FC algorithm with the genetic FA.

### 2.1 Electric vehicle recovery power allocation based on FC

Since the birth of electric vehicles, the range performance has always been a focus. To utilize this valuable energy to improve the endurance mileage, a system that can store the kinetic energy of a vehicle’s forward movement in some way and convert it into power for the vehicle when necessary is designed, namely, the brake energy recovery system [16,17]. The braking of fuel vehicles only consumes kinetic energy, while electric vehicles can recover kinetic energy, which is attributed to the extremely complex structure and basic principles of the electric vehicle BER system [18]. Accordingly, how to ensure the maximum BER while also ensuring the uniform distribution of vehicle energy and safe and smooth braking is an urgent technical problem. Therefore, the BER process of electric vehicles is subdivided and calculated. When the electric vehicle is in the continuous flow stage and preparing for braking, the energy feedback point of the first stage is shown in equation (1).


$$i\cdot R_{2}+E_{0}+L\frac{di}{dt}=0$$ (1)

In equation (1), *i* represents the amount of loop current at the energy feedback point. $E_{0}$ represents the current potential of the driver program. $R_{2}$ represents normal resistance. di represents the tiny line elements of this path. dt represents the transmission time of this path. *L* is the path from the reference position to the braking position. Therefore, when the current is in a reverse flow state and the electric vehicle is ready to brake, the energy feedback point calculation for the second stage is shown in equation (2).


$$i_{1}\cdot R_{2}+E_{1}+L\frac{di}{dt}=0$$ (2)

In equation (2), $i_{1}$ represents the reverse loop current at the energy feedback point. $E_{1}$ represents the reverse current potential of the driver program. The second stage of BER can maximize the braking energy return from the first stage. When the current flows back into the battery, the power switch is turned off. The energy feedback point of the third stage is calculated in equation (3).


$$i_{3}=\frac{U_{L}-E_{3}}{R_{2}}+\left(I_{on}-\frac{U_{L}-E_{3}}{R_{2}}\right)e^{-\left(R2/L\right)t}$$ (3)

In equation (3), $i_{3}$ represents the amount of supplementary circuit current at the energy feedback point. $U_{L}$ represents the voltage of the battery at this time. $E_{3}$ represents the current potential at both ends of the driver program. *e* represents the electrical energy at the energy feedback point. When the power switch is continuously turned off, the total amount of recovered braking energy during this period is shown in equation (4).


$$W=\int_{0}^{T_{off}}E\cdot i\cdot dt$$ (4)

**Fig 1:**
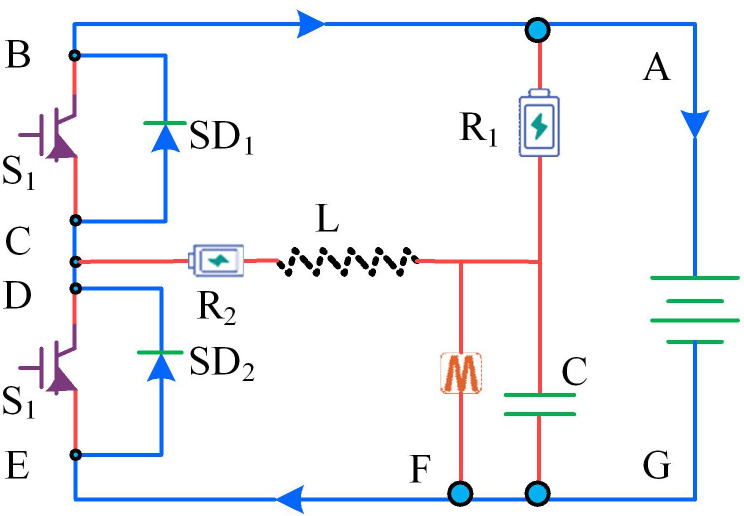
The flow of braking current when the electric vehicle is powered off.

In equation (4), *W* represents the total amount of recovered electricity. $T_{off}$ represents the power off time. The direction of energy recovery current for electric vehicles at this time is shown in Fig 1.

In addition to controlling and calculating the braking current, the power lithium battery state of electric vehicles is estimated. The FC is used for estimation. FC is an intelligent control technology widely used in industrial production. It is based on fuzzy set theory and uses language variables and fuzzy reasoning systems to describe an intelligent control technology [19]. FC is based on human experience and knowledge to establish a complete mathematical model. Then, the model is described using computer language and the system is controlled through programming [20]. The calculation process of the FC algorithm has three steps. The first is to blur the input, the second is to use a fuzzy rule library and fuzzy inference, and the last step is to output after deblurring. The deblurring calculation is shown in equation (5) [21].


$$K=\frac{\sum_{i=1}^{27}\beta_{i}K_{i}}{\sum_{i=1}^{27}\beta_{i}}$$ (5)

**Fig 2:**
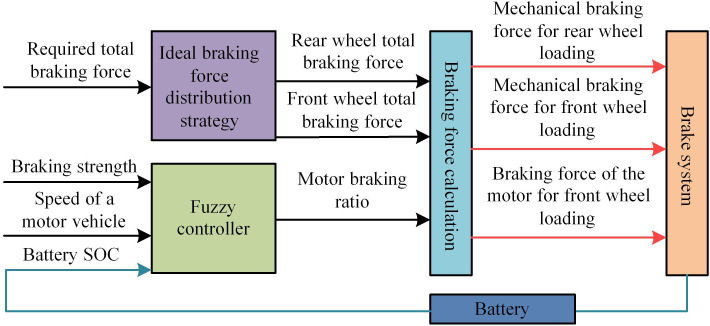
Schematic diagram of electric vehicle recovery power distribution system based on FC.

In equation (5), $\beta_{i}$ represents the fuzzy output under any rule. $K_{i}$ represents the membership function of the output value. *K* represents the braking energy distribution coefficient. The research aims to redistribute the braking force of electric vehicles through FC, ensuring driving safety and stability while recovering the maximum braking energy. The control system of electric vehicles is limited by various factors during braking energy feedback, among which the State of Charge (SOC), vehicle speed, and braking intensity have the greatest impact. SOC is a very important parameter that reflects the current charging status of the battery, usually expressed as a percentage. SOC has a crucial impact on the performance, safety, battery life, and user experience of electric vehicles. The study takes these variables as the three input parameters of the fuzzy controller. The output value is the proportion of the motor braking force to the total braking force allocated to the front axle. The ultimate goal is to maximize the participation of regenerative braking force in braking while ensuring smooth braking to efficiently recover braking energy. The proposed electric vehicle recovery power distribution system based on FC is displayed in Fig 2.

From Fig 2, the recovery power distribution system for electric vehicles based on FC algorithm is mainly composed of ideal braking force distribution strategy, fuzzy controller, braking force calculation, braking system, and lithium battery. Both the ideal braking force distribution strategy and the fuzzy controller require all data to be used for braking force calculation and reasonable allocation of braking energy [22]. From Fig 2, the inter axle distribution involves the distribution of braking force between the front and rear axles. The electromechanical distribution involves the distribution of mechanical braking and motor braking force. If the braking force of the front axle is too strong, it may cause instability of the vehicle. In this case, the distribution of motor braking force is adjusted to ensure the braking balance. The distribution between axles needs to meet certain safety regulations to ensure good braking performance of vehicles on roads with different adhesion coefficients. The electromechanical distribution needs to be optimized within these safety requirements to achieve effective energy recovery.

### 2.2 Control strategy based on improved genetic FA

There are many problems in the operation of the electric vehicle regenerative power distribution system based on the FC algorithm, such as algorithm rules of the fuzzy controller being more subjective and often unable to achieve optimal control effects. Therefore, based on the FC algorithm, the study aims to optimize the deep control strategy by maximizing the recovery of braking energy. The study categorizes the braking conditions of electric vehicles into three types: emergency braking, braking on long downhill slopes, and moderate to light braking, and uses these three braking conditions as the basis for the distribution strategy of front and rear braking force. Wheel lock up refers to the situation where the wheels are completely locked and stop rotating during the braking process, causing the vehicle to lose direction control. When the wheel locks up, the friction between the tire and the road surface changes from rolling friction to sliding friction, which greatly weakens the friction coefficient of the tire and reduces braking force. Unlike wheel locking, wheel braking refers to the process of applying braking force to the wheels through a braking system to slow down or stop the vehicle. The distribution of braking force when the front wheels are first locked is shown in equation (6).


$$F_{xb2}=\frac{L-\varphi h_{g}}{\varphi h_{g}}F_{xb1}-\frac{Gb}{h_{g}}$$ (6)

In equation (6), $F_{xb1}$ represents the ground braking force of the front wheels. $F_{xb2}$ represents the ground braking force of the rear wheels. *φ* represents the road adhesion coefficient, $h_{g}$ represents the height of the center of mass. *b* represents the distance from the center of mass to the rear axle. *L* represents the wheelbase. *G* represents the gravity, *m* represents the weight of electric vehicles. *g* represents the gravitational acceleration during vehicle braking. *z* represents the braking force. The calculation condition for equation (6) is low-intensity braking, with the front wheels bearing all braking force and prioritizing the use of motor braking force. The braking intensity at a certain point during the braking process is shown in equation (7).


$$\{\begin{array}{l} 0.2468F_{xb1}-271.48=0\\ F_{xb1}=mgz_{A_{2}} \end{array}$$ (7)

In equation (7), $z_{A_{2}}$ represents the braking intensity at the $A_{2}$ braking point, which is moderate. Therefore, the energy distribution calculation of rear wheel braking force for electric vehicles is shown in equation (8).


$$\{\begin{array}{l} F_{xb2}=0.2468F_{xb1}-271.48\\ F_{xb2}+F_{xb1}=mgz_{A_{2}} \end{array}$$ (8)

In equation (8), $F_{xb2}$ represents the ground braking force of the rear wheels. To ensure braking safety, the research combines the above calculation equation to adjust the distribution strategy, as shown in equation (9).


$$\{\begin{array}{l} F_{xb2}-1064=0.1251(F_{xb1}-5999)\\ F_{xb2}+F_{xb1}=mgz \end{array}$$ (9)

**Fig 3:**
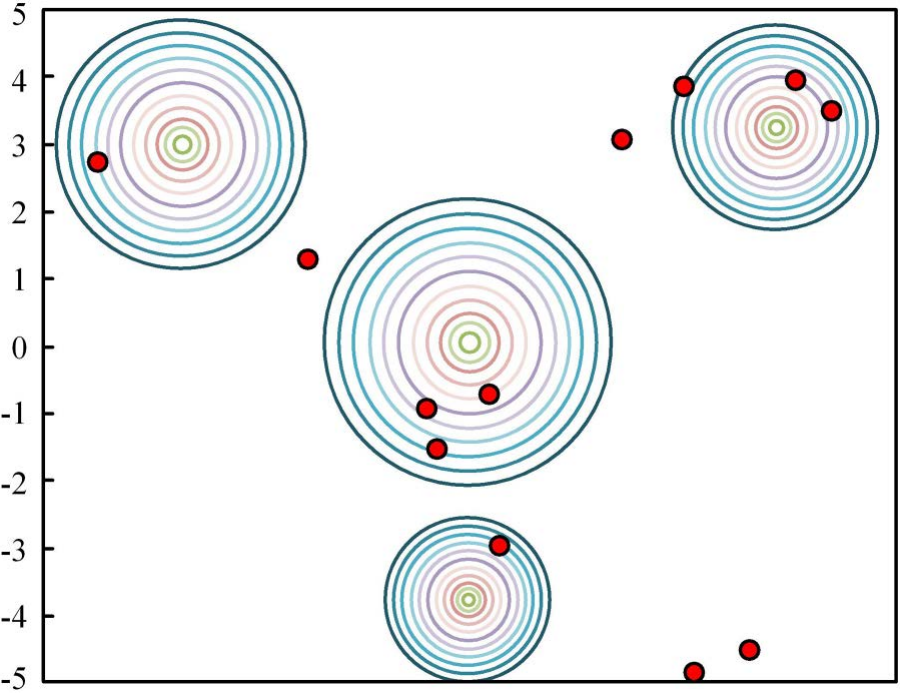
Schematic diagram of firefly initialization operation logic for FA.

In equation (9), when $z>z_{c}$ occurs, the front and rear braking forces are in high-intensity emergency braking. The optimized front and rear wheel braking force distribution strategy can be determined, which can set the braking recovery target to the maximum extent to optimize the working range of the braking system. Meanwhile, the FA is also adopted, which is a meta-heuristic algorithm based on the flickering behavior of fireflies. FA has been widely applied in many fields due to its superior performance in feature extraction, clustering, and other problems compared with other traditional algorithms [23]. In a randomly distributed 2D plane, the position of each “firefly” is expressed as $I(n)=f(x_{n},y_{n})$. Its initialization logic is shown in Fig 3 [24,25].

The three principles of the FA are highly compatible with the braking energy control. These three principles are that all energy points in the system attract each other, and points with stronger energy attract low-energy points to gather. The optimal solution is determined based on the intensity of energy [26]. Combined with FA, the strongest energy point for electric vehicles is displayed in equation (10).


I(x)∝f(x) (10)

In equation (10), I(x) represents the point of luminous intensity. f(x) represents the objective function. The braking energy is absorbed during transmission, so that the energy intensity is not conserved. The change calculation is shown in equation (11).


$$I(r)=I_{0}e^{-\gamma r^{2}}$$ (11)

In equation (11), $I_{0}$ represents the initial intensity of braking energy transmission. *γ* represents the absorption coefficient of energy during transmission. *r* represents the distance between two energy points. I(r) represents the intensity change during the transmission of braking energy. Between energy points that are close in distance, weaker energy is attracted by stronger energy points. The attraction is shown in equation (12).


$$\beta (r)=\beta_{0}e^{-\gamma r^{2}}$$ (12)

In equation (12), $\beta_{0}$ represents the initial attractive force of braking energy transmission, which is set to 1. $\beta_{0}$ also represents the attraction at the light source of fireflies in the FA. The reason for setting the attraction to 1 is to simplify the calculation and ensure that the movement in the algorithm is based on relative brightness differences rather than absolute brightness values. When the distance between fireflies is 0, the attraction is 1. As the distance increases, the attraction will be adjusted according to equation (12) [14,27]. The position of the energy point changes after being attracted. The position calculation is shown in equation (13).


$$x_{i}^{t+1}=x_{i}^{t}+\beta_{0}e^{-\gamma r^{2}}\left(x_{j}^{t}-x_{i}^{t}\right)+\alpha^{t}(rand-\frac{1}{2})$$ (13)

**Fig 4:**
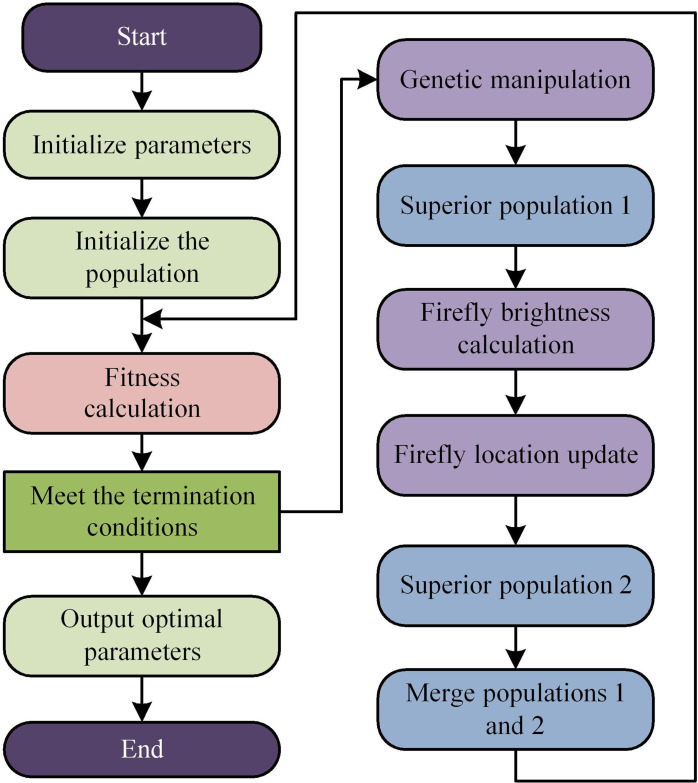
Schematic diagram of the operation logic of the improved genetic algorithm.

In equation (13), $x_{i}$ represents the energy point position *i*. $x_{j}$ represents the energy point position *j*. *α* represents a random parameter. *t* represents the evolutionary algebra. However, FA has a strong dependence on individual energy points, and excellent individuals can determine the direction [28,29]. Therefore, the genetic algorithm is introduced to improve FA. Genetic algorithm can use its excellent global search ability to search for outstanding individuals in the entire energy collection, which facilitates the operation and calculation of FA. The fusion algorithm has strong parallel processing capabilities and can handle multiple individuals simultaneously. It also has strong adaptive search capability, which helps improve the computational efficiency of the algorithm. The improved FA operation logic is shown in Fig 4.

### 2.3 Brake energy recovery control model integrating FC and improved FA

**Fig 5:**
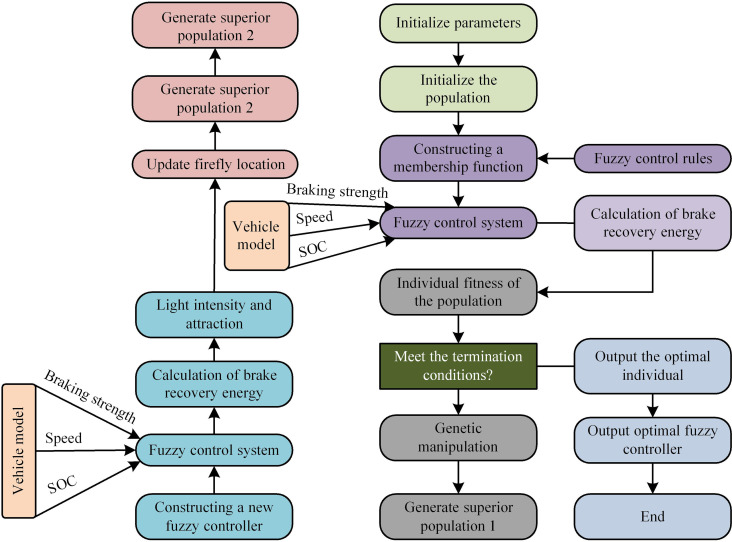
The process of FC-FA.

After introducing genetic FA to optimize the braking energy recovery control strategy of electric vehicles, the strategy is further modeled. The study combines FC with improved FA to form a fused FC-FA electric vehicle braking energy recovery control model. The FC-FA model not only includes the professional experience evaluation section of the FC algorithm, but also optimizes the decoding time and efficiency of the FC algorithm by combining it with the FA. The FC-FA control logic is shown in Fig 5.

From Fig 5, the model first initializes the population of braking energy in order to construct the membership function and form a preliminary FC controller. The controller can initially calculate the braking recovery energy and comprehensively observe the individual fitness of the population for these energies. If the fitness meets the requirements, the iteration will be stopped. If it is not met, further genetic operations are required to search for the optimal population in the overall energy set and build a fuzzy control system with a new excellent population. Finally, all energy points of the system are affected by the FA, with weaker energy being attracted by stronger energy, improving the efficiency of BER [30]. The Mean Absolute Error (MAE) is used to evaluate local energy points, as calculated in equation (14).


$$MAE=\frac{1}{n}\sum_{i=1}^{n}\left|\hat{y}i-yi\right|$$ (14)

In equation (14), *n* represents adjustable parameters. $\hat{y}i$ represents predicted values. yi represents true values. The smaller the MAE, the better the accuracy of the BER control model [31]. In addition, the Root Mean Squared Error (RMSE) is also used to assess the overall model performance, as calculated in equation (15).


$$RMSE=\sqrt{\sum {\left(y_{i}-y_{i}^{\prime }\right)}^{2}/n}$$ (15)

In equation (15), RMSE represents the squared expected value of the error. If the result is equal to 10, the average difference between the regression effect and the true value is 10 [32]. In addition to error control, the fuzzy control rules also affect the results of BER control throughout the entire model. The proposed fuzzy control rule strives to use regenerative braking force of the motor as much as possible while ensuring the stability and safety of vehicle braking, so that the BER performance can be fully utilized. The rule making principle of fuzzy control can be divided into four points. First, when the braking intensity is low, the motor braking force should be used. When the motor braking force cannot meet the braking demand, hydraulic compression power is used as a backup supplement. When the braking intensity is high or even reaches an emergency braking state, the braking force of the motor gradually decreases or is not used at all. Secondly, when the vehicle speed is fast, that is, when the available kinetic energy is large during the braking process, the proportion of motor braking force is increased as much as possible. Thirdly, when the battery SOC is in a high state or even exceeds 95%, the proportion of motor braking force should be gradually reduced or even the regenerative braking should be completely stopped, and the BER function should be turned off. Fourth, when the braking intensity, vehicle speed, and SOC are all in the middle of the fuzzy domain, the proportion of motor braking force should be appropriately increased to fully utilize the regenerative braking force. The established fuzzy rules are shown in Table 2.

**Table 2:** Control rules for braking energy of electric vehicles.

Number	Z	SOC	V	K	Number	Z	SOC	V	K	Number	Z	SOC	V	K
1	L	H	L	L	10	L	M	L	L	19	L	L	L	L
2	L	H	M	L	11	L	M	M	H	20	L	L	M	H
3	L	H	H	L	12	L	M	H	H	21	L	L	H	H
4	M	H	L	L	13	M	M	L	L	22	M	L	L	L
5	M	H	M	L	14	M	M	M	M	23	M	L	M	M
6	M	H	H	L	15	M	M	H	M	24	M	L	H	M
7	H	H	L	L	16	H	M	L	L	25	H	L	L	L
8	H	H	M	L	17	H	M	M	L	26	H	L	M	L
9	H	H	H	L	18	H	M	H	L	27	H	L	H	L

**Fig 6:**
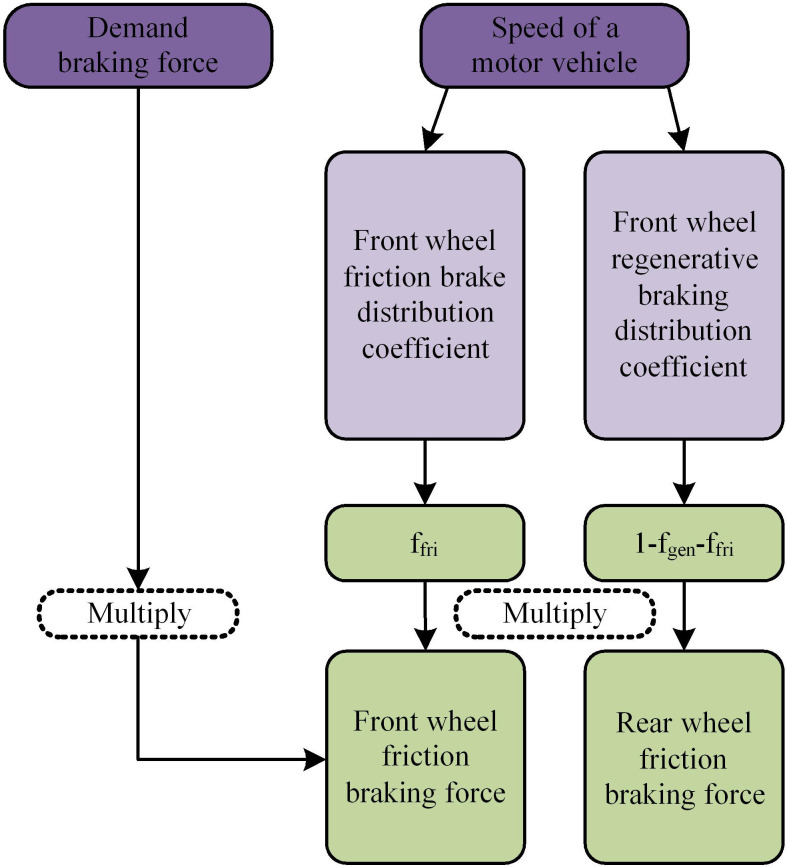
Allocation method of FC-FA model in ADVISOR software.

After mastering the fuzzy control rules, the model can be applied to simulate the operation software of electric vehicles for braking energy simulation. The Advanced Vehicle Simulator (ADVISOR) software is applied. This software needs to be developed in MATLAB and SIMULINK software environments. It is more suitable for research institutes and universities. In ADVISOR software, the FC-FA electric vehicle BER control model can be used for simple braking force distribution. The allocation method of FC-FA model in ADVISOR software is displayed in Fig 6.

From Fig 6, the FC-FA allocation method in ADVISOR software is determined by both the required braking force and the vehicle speed, which includes the front wheel friction braking allocation coefficient and the front wheel recovery braking allocation coefficient. The required braking force and vehicle speed need to be multiplied separately to obtain the front axle friction braking force and the rear axle friction braking force, respectively.

## 3. Results

To verify that the designed BER model has better control performance, algorithm validation and model simulation experiments are conducted. The experiment first verifies the effectiveness and control advantages of the FC algorithm, followed by the performance comparison before and after FA optimization. Finally, the BER of FC-FA is compared.

### 3.1 Experimental analysis of fuzzy algorithm for braking energy control of electric vehicles

To verify the effectiveness of the proposed FC rule in controlling the braking energy of electric vehicles, experiments are conducted. The experiment selects the driving speed, braking intensity, and SOC of electric vehicles for comparison, among which SOC can determine the function of the fuzzy control rules, divide the characteristics of software and hardware, and pay attention to the design. The experiment first determines an initial speed of 10Km/h, a slight braking intensity of 0.1, a moderate intensity of 0.3, and a maximum intensity of 0.7. The initial SOC is determined to be 70%. Based on the above data support, Table 3 displays the parameter changes in the braking process.

**Table 3:** Statistics of braking process parameters.

Initial speed of braking/Km/h	Braking time/s	Braking distance/m	Terminate SOC	Recovered braking energy/kJ	Energy changes at the battery end/kJ	Braking energy recovery rate
10	0.95	1.31	70.01%	9.24	3.01	32.58%
30	3.01	13.01	70.06%	89.60	33.00	36.85%
60	5.67	48.31	70.20%	184.96	123.54	66.38%
90	8.55	106.3	70.55%	420.38	359.9	85.61%

**Fig 7:**
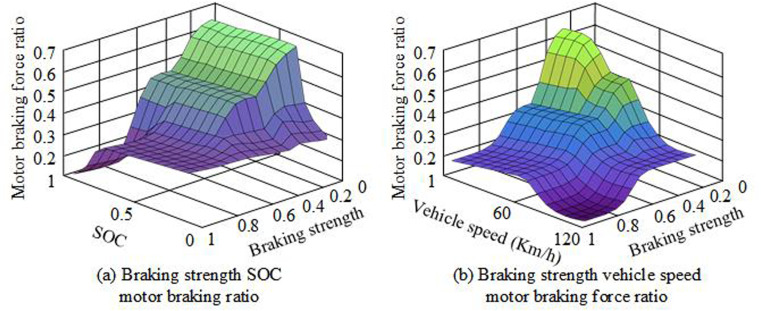
Surface recognition diagram of the effect of FC control algorithm on the braking intensity, moving speed, and SOC index of electric vehicles.

In Table 3, as the initial braking speed gradually increased, the braking time, braking distance, SOC, and recovered braking energy also gradually increased. When the initial braking speed was 10km/h, the braking time was 0.95s. The vehicle moved 1.31m to stop, and the SOC value was 70.01%. Under these data conditions, the recovered braking energy through the FC algorithm was 9.24KJ, with the smallest energy change at the battery end and a low BER efficiency of 32.58%. When the initial braking speed increased to 90Km/h, the braking time consumed increased to 8.55s. During this period, the vehicle moved 106.3m from braking to parking, and the SOC value was 70.55%. Under these data conditions, the recovered braking energy through FC algorithm was 420.38KJ, with the largest change in energy at the battery end and a low BER efficiency of 85.61%. This indicates that the FC rule can control the BER efficiency and amount by calculating the vehicle speed, which also demonstrates the effectiveness of the fuzzy rule. This result is relatively close to the research findings obtained in reference [15]. Subsequently, the FC rule is input into the fuzzy controller to verify the effectiveness of the FC in surface recognition of the braking intensity, moving speed, and SOC of electric vehicles, as displayed in Fig 7.

**Fig 8:**
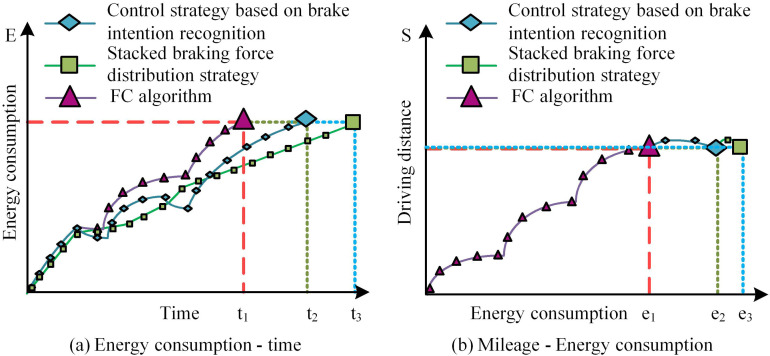
Comparison of the control efficiency of electric vehicle braking energy recovery between the control algorithm and comparative methods.

In Fig 7 (a), the motor braking ratio of electric vehicles was closely related to the braking intensity. As the SOC value increased, the motor braking ratio also increased, and the restriction state was released. When SOC was greater than or equaled to 1, the maximum proportion of motor braking force reached its maximum value at a braking intensity of 0.2, which was 0.68. The closer the braking intensity is to 1, the closer the motor braking ratio is to 1. In Fig 7 (b), the motor braking ratio of electric vehicles was closely linked to the braking speed of electric vehicles. As the vehicle speed increased, the motor braking ratio also changed accordingly. When the braking speed was below 20Km/h, the motor braking ratio maintained between 0.1-0.25. When the braking speed was below 20Km/h, the motor braking ratio was maintained between 0.1-0.25. The maximum value of the motor braking force ratio reached 0.61 when the braking intensity was 1. When the braking intensity approached 0.4, the motor braking ratio reached its maximum, at 0.68. This indicates that the FC rule input into the fuzzy controller can verify the effectiveness of the FC on the braking intensity, moving speed, and SOC of electric vehicles. Then, a comparative experiment is conducted to verify the control effect of FC on BER. The experiment introduced a control strategy based on brake intention recognition and a stacked brake force distribution strategy as comparative methods, and all three methods were tested under optimal conditions. The former identifies the driver’s braking intention and braking intensity, and formulates a reasonable braking force distribution strategy. The latter can be classified into stacked types based on the hardware system scheme for regenerative braking energy recovery. This strategy requires precise monitoring and analysis of pedal force and pedal displacement signals. The conditions for the comparative experiment are consistent road conditions, braking intensity, and driving speed. The maximum speed is 150km/h, the acceleration time is 12 seconds, and the mileage is 300km. The comparison results are shown in Fig 8.

In Fig 8 (a), the FC braking energy recovery strategy completed braking energy recovery in a shorter time with the same braking energy consumption. Compared with electric vehicles that do not use FC energy recovery strategy in the comparative methods, controlling to the former’s consumption level requires longer braking time support. In this situation, electric vehicles based on brake intention recognition control strategies often cannot reserve more time and braking distance when encountering high traffic flow and frequent braking needs. This also indicates that electric vehicles using the FC strategy can more effectively control the braking energy consumption and help detect the contribution of braking energy. From Fig 8 (b), electric vehicles using the FC braking energy recovery strategy traveled more miles and recovered more braking energy under the same braking energy consumption. Electric vehicles that do not use the FC braking energy recovery comparison strategy consume more energy during braking. Faced with complex driving conditions, its applicability is weak. This indicates that electric vehicles using the FC strategy can effectively control the braking energy consumption and help detect the contribution of braking energy. In summary, applying FC control strategy to electric vehicle BER is effective.

### 3.2 Performance analysis of improved FA

The efficiency of battery side BER before and after improving FA is compared. Before starting the experiment, the basic parameters related to the algorithm are set, as shown in Table 4.

**Table 4:** Relevant parameter settings for the algorithm proposed in the experiment.

Algorithm	Parameter	Description	Recommended value
Genetic Algorithm	Population size	Number of individuals in each generation	100
	Crossover probability	Probability of cross operation	0.7
	Mutation probability	Probability of performing mutation operations	0.01-0.05
Firefly Algorithm	Firefly count	The number of fireflies in the group	20-50
	Maximum Number Of Iterations	The maximum number of iterations for the algorithm to run	100-200
	Attraction coefficient	Attenuation coefficient of attraction between fireflies	0.1-0.5
	Local search parameters	Control local search intensity	0.2
	Randomly moving parameters	Control the intensity of random movement of fireflies	0.1-0.5

**Fig 9:**
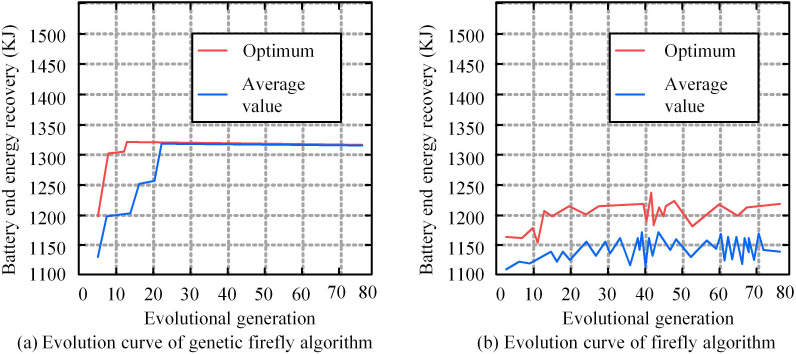
Comparison of battery terminal energy recovery before and after FA optimization.

The experiment optimized the FA in Matlab script files and compared the computational efficiency of 80 iterations before and after optimization. The premise of energy recovery at the battery end ensured good SOC state and battery temperature. To improve the sensitivity of the algorithm, the energy recovery at the battery end is uniformly set to 1500KJ, which is the recovery amount under the standard energy feedback mode in common electric vehicle models. The comparison between the best value and the average value is shown in Fig 9.

**Fig 10:**
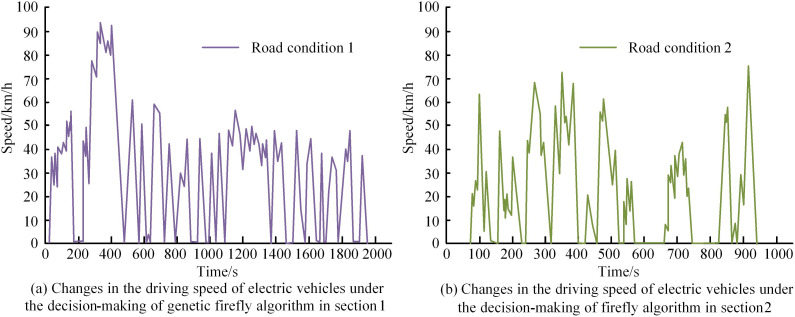
Comparison of braking energy recovery under two different road conditions.

In Fig 9 (a), when the optimized genetic FA reached 22 iterations, the average energy recovery at the battery end tended to converge, with a value of 1323KJ. After 22 iterations, the value remained constant. The optimal value of battery end BER occurred at 13 iterations, with a value of 1325KJ, and remained constant after 13 iterations. When the experimental iteration reached a certain termination condition, the value converged, indicating that the algorithm parameters input by the research met the experimental needs. In numerical calculations, algorithms with high stability are less sensitive to input errors, and global convergence means that no matter where the algorithm starts, it can eventually converge to the global optimal solution. This indicates that the improved genetic FA can consume less iterations, achieve stable and accurate control effects, and have high computational efficiency. In Fig 9 (b), the FA without genetic algorithm optimization had poor control effect on the battery end BER, and no stable value appeared. The maximum average value appeared at 43 iterations, at 1175KJ, and the lowest value appeared at the third iteration, at 1113KJ. The optimal value of battery end BER occurred at the 43rd iteration, with a value of 1248KJ, and the lowest value occurred at the 11th iteration, with a value of 1152KJ. This indicates that the FA without genetic algorithm improvement has poor control effect on battery end BER. Subsequently, the experiment is conducted to verify the braking energy recovery using the improved FA under different road and vehicle speed conditions. To improve the persuasiveness of the experiment, two different real-life traffic scenarios are set up: (1) Roads in the city center area, where the traffic flow can reach over 200 vehicles in 15 minutes during peak hours. The average speed is 20-30km/h, with an average of 2-3 traffic lights per kilometer. In areas with high commercialization, pedestrian density can reach 1-2 people per square meter. The roads in suburban residential areas have a traffic flow of less than 50 vehicles within 15 minutes, with an average speed of 50-70 km/h. There is only one or no traffic light within one kilometer, and there are very few pedestrians. The proposed method is applied to these two real road conditions for simulation analysis. The comparison results are shown in Fig 10.

**Fig 11:**
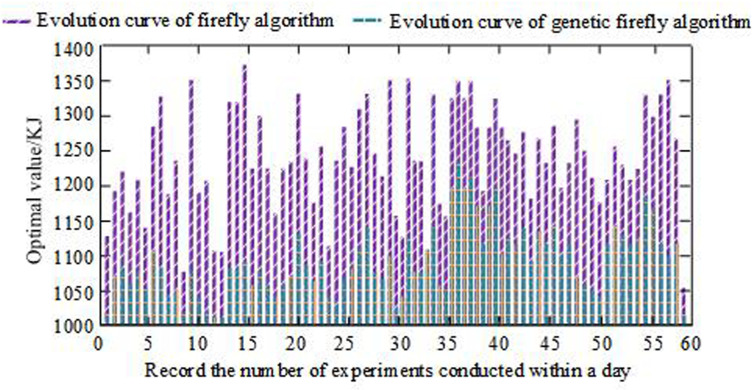
Energy recovery of electric vehicle braking before and after FA optimization.

In Fig 10 (a), in road condition 1, due to the high traffic volume and frequent braking cycles, the FA strategy was used to recover more braking energy. Between 200s-440s, the vehicle traveled at the fastest speed of 95km/h. This indicates that the brake recovery is lower and the braking frequency is lower. Electric vehicles driving at low speeds have less kinetic energy and less recoverable energy, but the proposed method can adapt to complex motor and mechanical braking, achieving effective energy recovery while ensuring braking stability and comfort. In Fig 10 (b), road condition 2 had a smaller traffic flow and fewer braking cycles compared with the road condition 1. Therefore, the BER based on the FA strategy was less. Between 900s-1000s, the vehicle traveled the fastest, at 75km/h, which also means that there is a greater amount of braking recovery per cycle and a lower braking frequency. From the BER under two different road conditions, the BER of electric vehicles is directly related to road traffic flow, vehicle speed, and motor participation. The BER is highest when braking is frequent, the vehicle speed is slow, and the driving time is longer. This also proves that the proposed method can determine the ratio of mechanical braking force and regenerative braking force according to different braking intensities. The proposed method is applicable to various braking conditions, can adaptively adjust the distribution of braking force, improve energy recovery rate, and ensure braking stability. Before and after optimization, FA is applied to conduct actual driving tests on electric vehicles. The BER of electric vehicles driving in road condition 1 and road condition 2 within one day is compared. The results are shown in Fig 11.

In Fig 11, the overall BER of the FA combined with genetic algorithm was much higher than that of the ordinary FA control. After optimizing the FA, the optimal value of BER appeared at the 15th iteration, with a value of 1325.30KJ, and the lowest value appeared at the 58th iteration, with a value of 1050.20KJ. The iteration difference between the optimal and minimum values is due to changes in the peak and valley periods of traffic flow. The period before and after 15 iterations is the morning rush hour, while the period before and after 50 iterations is around 1am, with the minimum traffic flow. The overall BER of ordinary FA was lower than that of genetic FA. Influenced by the ordinary FA, the optimal value of BER appeared in the 34th iteration, with a value of 1250.78KJ. The lowest value appeared in the 13th iteration, with a value of 1007.10KJ. This indicates that the unimproved FA cannot recover more energy during periods of high braking frequency. More experiments need to be conducted on more electric vehicles to obtain accurate values, which affects the consistency between energy recovery and braking frequency and reduces recovery efficiency. Comparing the results of this study with the new fuzzy sliding mode control scheme proposed in reference [12], the optimized genetic FA algorithm can achieve higher braking energy recovery efficiency in fewer iterations. This indicates that the genetic FA can converge to the optimal solution faster and recover more braking energy when dealing with electric vehicle braking energy recovery problems, which is of great significance for improving the energy utilization efficiency and extending the driving range of electric vehicles.

### 3.3 Simulation experiment analysis of energy recovery control for electric vehicle braking

**Fig 12:**
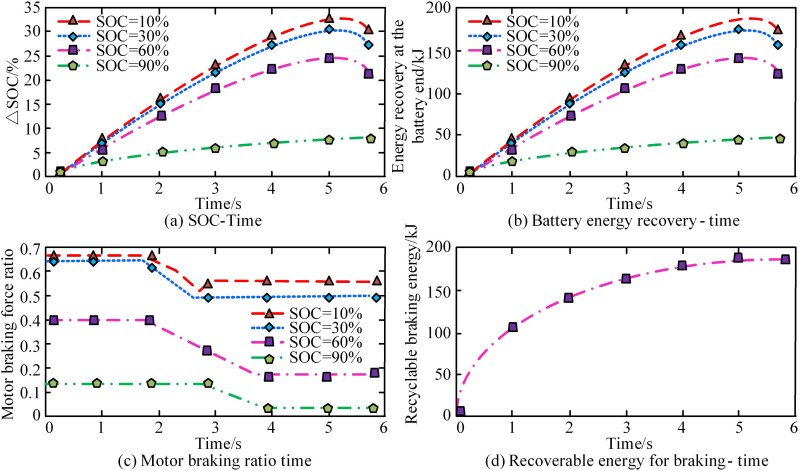
Parameter changes in the braking energy recovery process of the FC-FA.

To verify the effectiveness of the designed FC-FA electric vehicle braking energy recovery control model, simulation experiments are conducted. The experiment introduced the CLTC-P operating condition electric vehicle braking data as the analysis basis, and analyzed the SOC, battery terminal recovered energy, motor braking force ratio, and recoverable braking energy of the FC-FA model. The simulation experiment also combines six types of electric vehicles, including Tesla Model 3, BYD’s pure electric series, GAC Aion Y, Xiaopeng P7, NIO ES8, and Wuling Hongguang MINI EV, as targets for extracting actual braking data. The experiment uses MATLAB/Simulink as the simulation environment, combined with dSPACE real-time simulation system for hardware in the loop simulation. The results are shown in Fig 12.

In Fig 12 (a), the recovered braking energy of the brake motor varied at different SOC values. The FC-FA model had smaller impacts on SOC as the SOC value increased within the same time frame. In Fig 12 (b), when the SOC value was 10%, the total amount of recovered energy at the battery end was the highest, while when the SOC value gradually increased to 90%, the total amount of recovered energy at the battery end decreased. In Fig 12 (c), when the SOC value was 10%, the proportion of motor braking force was the highest. When the SOC gradually increased to 90%, the proportion of motor braking force decreased. In Fig 12 (d), the recoverable energy of braking gradually increased with time. After 5s, the growth trend of braking energy was gentle, at 180KJ. This indicates that the FC-FA control model can accurately control the BER under the introduced operating conditions, which maximizes the recovery amount. The SOC changes and battery energy output values under FC-FA model control are verified through practical application. Three traditional control methods, including parallel allocation strategy, non-braking energy recovery strategy, and fuzzy control strategy, are introduced as comparative methods. The verification results are shown in Table 5.

**Table 5:** SOC changes and actual application verification results of battery terminal energy output values under four control methods.

Data indicators	FC-FA	Parallel allocation strategy	Non-braking energy recovery strategy	Fuzzy control strategy
Total energy during braking process/kJ	3550	/	/	/
Recyclable braking energy/kJ	3330	/	/	/
SOC reduction amount/%	12.34	13.34	13.1	12.79
Energy recovery at the battery end/kJ	1580	1330	1347	1032
Energy consumption at the battery end/kJ	7311	7452	7654	7980
Driving distance/km (SOC decreased by 10%)	12.9	11.78	11.83	11.98
Braking energy recovery rate (%)	52.1	35.62	43.50	48.72
Comprehensive evaluation value (%)	40.28	34.25	28.69	38.65

In Table 5, the four control methods had similar control effects on the battery terminal SOC value under CLTC-P operating conditions. The SOC reduction of the FC-FA model was 12.44%, while the reduction values of the parallel allocation strategy, non-braking energy recovery strategy, and fuzzy control strategy were 13.34%, 13.1%, and 12.79%, respectively. These values are higher than those of the proposed method, indicating that the proposed method can more accurately control braking energy and ensure the battery life of electric vehicles. In terms of energy recovery at the battery end, the proposed method recovered 1580kJ of energy, and the fuzzy control strategy had the smallest recovery amount, with a recovery value of 1032kJ. In terms of energy consumption at the battery end, the proposed method consumed the least amount of energy, with a value of 7311kJ. The fuzzy control strategy consumed the most energy, with a value of 7980kJ. This indicates that electric vehicles using the proposed method can travel longer distances at the same SOC reduction rate. The FC-FA model achieved a BER rate of 52.1% in practical applications. The recovery rates of parallel allocation strategy, non-braking energy recovery strategy, and fuzzy control strategy were 35.62%, 43.50%, and 48.72%, respectively. The BER rate of these three traditional control methods is lower than that of the proposed method. In practical applications, the method proposed in the study has the advantage. Based on the above data, the FC-FA electric vehicle BER control model can effectively control the amount of energy recovery, ensuring maximum recovery while ensuring vehicle battery durability and driving stability. Finally, the experimental results are compared with benchmark methods, including state-of-the-art models, as shown in Table 6.

**Table 6:** Summary of research results.

Indicators/Methods	Braking energy recovery efficiency (%)	Recovered energy (KJ)	Total amount of energy recovered at the battery end (KJ)	Comprehensive evaluation value (%)	Reference
Genetic FA (optimal)	/	132530	/	/	Zare M et al [14]
Genetic FA (minimum)	/	1050.20	/	/	
Parallel strategy	35.62	/	1330	34.25	Armenta-Déu C al [7]
No recycling strategy	43.50	/	1347	28.69	Guo B et al [10]
fuzzy control strategy	48.72	/	1032	38.65	Wang Y et al [13]
FC-FA	52.1	3550	1580	40.28	This study

## 4. Discussion and conclusion

The electric vehicle is an effective way to address energy and environmental problems, but their mileage has always been regarded as the biggest purchasing criterion. Therefore, how to ensure low-carbon endurance without affecting driving safety has become an urgent problem in the electric vehicle industry. Therefore, the study combined FC with FA to build an electric vehicle BER control model. The results showed that the recovered braking energy through the FC algorithm was 9.24KJ, with the smallest energy change at the battery end and a low BER efficiency of 32.58%. Under these data conditions, the FC algorithm controlled the BER to be 420.38KJ, with the largest change in energy at the battery end and a low BER efficiency of 85.61%. The overall BER of the FA combined with genetic algorithm was much higher than that of the ordinary FA control algorithm. Under the genetic FA, the optimal value of BER appeared at the 15th iteration, at 1325.30KJ. The lowest value appeared at the 58th iteration, at1050.20KJ. The overall BER of ordinary FA was lower than that of genetic FA. The FC-FA model had the highest total amount of recovered energy at the battery end when the SOC value was 10% within the same time period. Conversely, as the SOC value gradually increased to 90%, the total amount of recovered energy at the battery end decreased. The FC-FA control model can accurately control the BER and maximize the amount of recovery, which helps to improve the endurance of electric vehicles. However, the study only conducts data analysis on BER, which can be further implemented in practical operations to enhance the research value.

## 5. Limitations and future work

In practical driving scenarios, such as frequent start stop on congested urban roads, long-distance driving on highways, and driving under different weather and road conditions, the deployment and application of the proposed method will face some challenges. For example, integrating the FC-FA model into existing electric vehicle control systems may require significant adjustments to the vehicle’s hardware and software. The potential pathway for this challenge is to collaborate with automobile manufacturers to develop customized control units and software to ensure seamless integration of FC-FA models into existing systems of vehicles. Consumers may adopt a wait-and-see attitude towards new technologies, especially if they have doubts about their performance and reliability. This requires showcasing the advantages of the new system through educational marketing and providing a trial period, as well as continuously improving the technology through user feedback. In addition, pure electric vehicles need to comply with regulatory requirements for the distribution of braking force between the front and rear wheels during the braking process, which may limit the optimization of energy recovery. Therefore, in subsequent research, MATLAB can be used to build control strategy models and conduct joint simulations in automotive dynamics simulation software. This proves that the proposed braking energy recovery control strategy can effectively improve the range of hybrid electric vehicles.

## Supporting information

S1 Text Minimal data set. (DOCX)
