# Supplementary material for: Electric vehicle braking energy recovery control method integrating fuzzy control and improved firefly algorithm
Source: PLoS One. 2025 Mar 28;20(3):e0320537. doi: 10.1371/journal.pone.0320537 (PMC11952267; doi:10.1371/journal.pone.0320537)
Supplement: S1 Text — (DOCX) [file pone.0320537.s001.docx]

**The data in Figure 7**

| Number | Braking strength SOC motor braking ratio | | | Braking strength vehicle speed motor braking force ratio | | |
| --- | --- | --- | --- | --- | --- | --- |
|  | SOC | Braking strength | Motor braking ratio | Braking speed (Km/h) | Braking strength | Motor braking ratio |
| 1 | 0.1 | 0.1 | 0.1 | 10 | 0.1 | 0.1 |
| 2 | 0.1 | 0.2 | 0.68 | 10 | 0.2 | 0.15 |
| 3 | 0.1 | 0.4 | 0.68 | 10 | 0.4 | 0.25 |
| 4 | 0.1 | 0.6 | 0.8 | 10 | 0.6 | 0.3 |
| 5 | 0.1 | 0.8 | 0.9 | 10 | 0.8 | 0.35 |
| 6 | 0.5 | 0.1 | 0.2 | 20 | 0.1 | 0.15 |
| 7 | 0.5 | 0.2 | 0.7 | 20 | 0.2 | 0.2 |
| 8 | 0.5 | 0.4 | 0.7 | 20 | 0.4 | 0.3 |
| 9 | 0.5 | 0.6 | 0.85 | 20 | 0.6 | 0.4 |
| 10 | 0.5 | 0.8 | 0.95 | 20 | 0.8 | 0.45 |
| 11 | 1.0 | 0.1 | 0.3 | 30 | 0.1 | 0.2 |
| 12 | 1.0 | 0.2 | 0.68 | 30 | 0.2 | 0.25 |
| 13 | 1.0 | 0.4 | 0.7 | 30 | 0.4 | 0.35 |
| 14 | 1.0 | 0.6 | 0.85 | 30 | 0.6 | 0.45 |
| 15 | 1.0 | 0.8 | 0.95 | 30 | 0.8 | 0.5 |

**The data in Figure 8**

| Braking strength | Energy consumption - time | | Mileage - Energy consumption | |
| --- | --- | --- | --- | --- |
|  | FC braking energy recovery strategy | Comparison method | FC braking energy recovery strategy | Comparison method |
| Low | 0-45 minutes | 0-98 minutes | 0-100 miles | 0-50 miles |
| medium | 45-110 minutes | 98-180 minutes | 100-200 miles | 50-90 miles |
| high | 110-200 minutes | 180-240 minutes | 200-300 miles | 100-150 miles |

**The data in Figure 9**

| Epochs | Optimized genetic FA algorithm for battery end energy recovery | | Unoptimized FA algorithm for battery end energy recovery | |
| --- | --- | --- | --- | --- |
|  | Average Energy Recovery (KJ) | Best Energy Recovery (KJ) | Average Energy Recovery (KJ) | Best Energy Recovery (KJ) |
| 10 | 1202 | 1315 | 1126 | 1161 |
| 20 | 1252 | 1323 | 1125 | 1219 |
| 30 | 1323 | 1325 | 1143 | 1228 |
| 40 | 1323 | 1325 | 1152 | 1232 |
| 50 | 1323 | 1325 | 1175 | 1248 |
| 60 | 1323 | 1325 | 1168 | 1232 |
| 70 | 1323 | 1325 | 1154 | 1225 |
| 80 | 1323 | 1325 | 1147 | 1233 |

**The data in Figure 10**

| Speed/km/h | Energy recovery of road braking in urban central areas | | Energy recovery of road braking in suburban residential areas | |
| --- | --- | --- | --- | --- |
|  | Time (s) | Braking energy recovery (%) | Time (s) | Braking energy recovery (%) |
| 0 | 0.-200 | 0% | 0.-100 | 0% |
| 10 | 200-400 | 1% | 100-200 | 1% |
| 20 | 400-600 | 2% | 200-300 | 2% |
| 30 | 600-800 | 3% | 300-400 | 3% |
| 40 | 800-1000 | 4% | 400-500 | 4% |
| 50 | 1000-1200 | 5% | 500-600 | 5% |
| 60 | 1200-1400 | 6% | 600-700 | 6% |
| 70 | 1400-1600 | 7% | 700-800 | 7% |
| 80 | 1600-1800 | 8% | 800-900 | 8% |
| 90 | 1800-2000 | 9% | 900-1000 | 9% |
| 100 | 2000+ | 10% | 1000+ | 10% |

**The data in Figure 13**

| SOC(%) | Energy recovery of road braking in urban central areas  Energy recovery of road braking in suburban residential areas | | | |
| --- | --- | --- | --- | --- |
|  | Time (s) | Motor braking force ratio | Energy recovery at the battery end/kJ | △SOC/% |
| 10 | 0 | 0.66 | 49 | 7 |
| 20 | 1 | 0.65 | 45 | 2 |
| 30 | 2 | 0.64 | 88 | 7 |
| 40 | 3 | 0.57 | 92 | 5 |
| 50 | 4 | 0.55 | 109 | 6 |
| 60 | 5 | 0.22 | 142 | 5 |
| 70 | 6 | 0.19 | 49 | 7 |
| 80 | / | 0.15 | 50+ | 8+ |
| 90 | / | 0.06 | 50+ | 8+ |
